# Kynurenine, 3-OH-kynurenine, and anthranilate are nutrient metabolites that alter H3K4 trimethylation and H2AS40 *O*-GlcNAcylation at hypothalamus-related loci

**DOI:** 10.1038/s41598-019-56341-x

**Published:** 2019-12-24

**Authors:** Koji Hayakawa, Kenta Nishitani, Satoshi Tanaka

**Affiliations:** 1 Department of Toxicology, Faculty of Veterinary Medicine, Okayama University of Science, Imabari-shi, Ehime Japan; 2 Laboratory of Cellular Biochemistry, Department of Animal Resource Sciences /Veterinary Medical Sciences, The University of Tokyo, Tokyo, Japan

**Keywords:** Gene regulation, Epigenetics

## Abstract

Epigenetic mechanisms can establish and maintain mitotically stable patterns of gene expression while retaining the DNA sequence. These mechanisms can be affected by environmental factors such as nutrients. The importance of intracellular dosages of nutrient metabolites such as acetyl coenzyme A and *S*-adenosylmethionine, which are utilized as donors for post-translational modifications, is well-known in epigenetic regulation; however, the significance of indirect metabolites in epigenetic regulation is not clear. In this study, we screened for metabolites that function as epigenetic modulators. Because the expression of genes related to hypothalamic function is reportedly affected by nutritional conditions, we used a neural cell culture system and evaluated hypothalamic-linked loci. We supplemented the culture medium with 129 metabolites separately during induction of human-iPS-derived neural cells and used high-throughput ChIP-qPCR to determine the epigenetic status at 37 hypothalamus-linked loci. We found three metabolites (kynurenine, 3-OH-kynurenine, and anthranilate) from tryptophan pathways that increased H3K4 trimethylation and H2AS40 *O*-GlcNAcylation, resulting in upregulated gene expression at most loci, except those encoding pan-neural markers. Dietary supplementation of these three metabolites and the resulting epigenetic modification were important for stability in gene expression. In conclusion, our findings provide a better understanding of how nutrients play a role in epigenetic mechanisms.

## Introduction

Epigenetic mechanisms are a complex of heritable states that regulate gene expression. They involve modifications in chromatin structure, typically at histones, that do not alter DNA sequences but influence gene expression^1,2^. As it modulates gene expression, epigenetics influences the development of multiple human diseases, including cancer^3,4^. In contrast with DNA mutation, environmental factors could potentially reverse epigenetic phenomena. Therefore, researchers are increasingly interested in understanding how the environment (e.g., nutrient status) affects epigenetics^4–6^.

Many epigenetic processes involve enzymes that use small-molecule metabolites generated during cellular metabolism^7–9^. For example, threonine-dependent changes in the S-adenosylmethionine/S-adenosylhomocysteine (SAM/SAH) ratio influence tri-methylation levels on lysine in histone H3^10^. Additionally, elevated production of α-ketoglutarate (KG) from glutamine metabolism promotes histone and DNA demethylation to maintain a relaxed chromatin state^11^. Furthermore, histone acetylation depends on intermediary metabolism to supply acetyl-CoA in the nucleocytosolic compartment^12^. Another notable example is the hexosamine biosynthesis pathway, which integrates the metabolism of glucose, glutamine, acetyl-CoA, and uridine-diphosphate into UDP-N-acetyl-glucosamine (UDP-GlcNAc) synthesis. This process influences levels of protein *O*-GlcNAcylation^13,14^.

Although many metabolites (e.g., SAM, acetyl-CoA, and UDP-GlcNAc) directly engage in epigenetic modifications, other metabolites exert their effects indirectly. Our previous study demonstrated that the addition of *N*-acetyl-D-mannosamine (ManNAc) induces orexin neurogenesis from mouse and human pluripotent stem cells^15,16^. Additionally, ManNAc epigenetically alters *HCRT* (coding prepro-orexin peptide), suggesting that metabolites beyond those directly performing epigenetic modifications can also regulate epigenetic status. However, little is known about the potential crosstalk between metabolites and epigenetics.

Here, we aimed to explore intermediate metabolites that play a role in epigenetics. Nutrients and metabolic status are known to influence gene expression in hypothalamic neurons, including those involved in differentiation^16–18^. Thus, hypothalamic genes and neurons could be useful for monitoring epigenetic responses to nutrient fluctuations. For our investigation, we performed chromatin immunoprecipitation (ChIP)–quantitative PCR (qPCR) to determine the epigenetic status at 37 hypothalamus-linked loci in neurons derived from human-induced pluripotent stem cells (hiPSCs). In that analysis, we demonstrated the sensitivity of neural cells to various metabolites by focusing on histone H3 lysine 4 trimethylation (H3K4me3)^19,20^ and histone H2A serine 40 *O*-GlcNAcylation (H2AS40Gc)^21,22^, two epigenetic markers that are known for their potential to be altered by extracellular glucose concentrations. Our study will provide a better understanding of the role of nutrients in epigenetic regulation and the use of nutrients or food components in modifying epigenetic status.

## Results

### Kynurenine, 3-OH-kynurenine, and anthranilate exert epigenetic effects on hypothalamic neural genes

**Figure 1:**
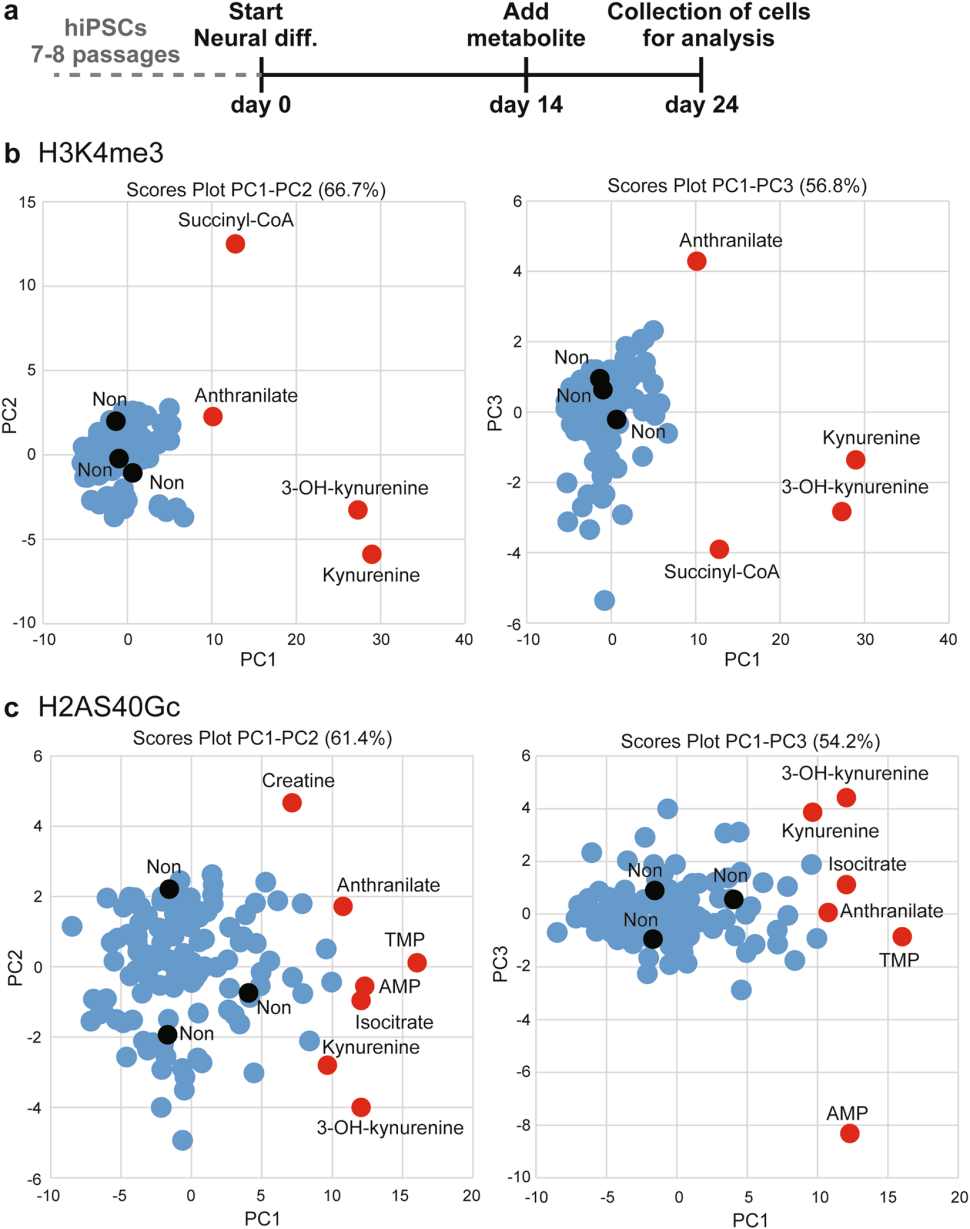
Epigenetic profile of H3K4me3 and H2AS40Gc in neurons cultured with selected metabolites. (**a**) Culture protocol for adding metabolites to human induced pluripotent stem cell (hiPSC)-derived neural cells. (**b**,**c**) Principal component analysis on ChIP-qPCR to determine H3K4me3 (**b**) and H2AS40Gc (**c**) levels. Red and black circles indicate significantly altered metabolites and non-treatment (Non), respectively.

The differentiated hiPSCs reportedly complete neural commitment at day 14 and then reach neurons expressing pan-neural markers such as TUBB3 and MAP2 at day 20^16,23^. To avoid suppressing the neural commitment of hiPSCs and to evaluate the effect of metabolites on neurons, 129 commercially available metabolites were individually added to the medium from day 14 until day 24 of culturing (Table 1 and Supplementary Table 1). Cells were collected on day 24 of culture to determine H3K4me3 and H2AS40Gc levels around the transcriptional start site (Fig. 1a). We selected the SDIA neural differentiation method to induce dopaminergic neurons from hiPSCs, rather than hypothalamic neurons^16,23^. This choice allowed us to better estimate the effects of metabolites on epigenetic activation at hypothalamic neural peptide-coding and differentiation-related loci. Before beginning epigenetic analysis, we confirmed via bright-field microscopy that for each metabolite, supplementation was not associated with a significant reduction in cells.

**Table 1:** Metabolites used in this study.

Class^*^	No. of metabolite
Nucleosides, nucleotides, and analogues	26
Pyrimidine nucleotides	1
5′-deoxyribonucleosides	1
Organic acids and derivatives	23
Organic oxygen compounds	17
Organic nitrogen compounds	4
Organic phosphoric acids and derivatives	1
Organic sulfonic acids and derivatives	1
Organoheterocyclic compounds	9
Organonitrogen compounds	2
Carboxylic acids and derivatives	9
Keto acids and derivatives	3
Lipids and lipid-like molecules	8
Diazines	2
Imidazopyrimidines	2
Indoles and derivatives	2
Benzenoids	3
Benzene and substituted derivatives	1
Azoles	1
Not classified	13

^*^Based on the Human Metabolome Database (http://www.hmdb.ca/).

We used principal component analysis (PCA) to analyze ChIP-qPCR data of H3K4me3 and H2AS40Gc at 37 loci (23 hypothalamic neuropeptide-coding genes and 14 neural differentiation-related genes) (Fig. 1b,c). The PC1-PC2 and PC1-PC3 plots revealed that treatment with four metabolites (succinyl-CoA, anthranilate, 3-OH-kynurenine, and kynurenine) resulted in markedly different H3K4me3 levels (Fig. 1b). Furthermore, the PC1-PC2 and PC1-PC3 axes respectively identified seven (creatine, anthranilate, TMP, AMP, isocitrate, kynurenine, and 3-OH-kynurenine) and six metabolites (3-OH-kynurenine, kynurenine, isocitrate, anthranilate, TMP, and AMP) that markedly changed H2AS40Gc levels (Fig. 1c). Notably, kynurenine, 3-OH-kynurenine, and anthranilate were common in both types of histone modification. Therefore, subsequent analyses focused on the effects of these three metabolites.

**Figure 2:**
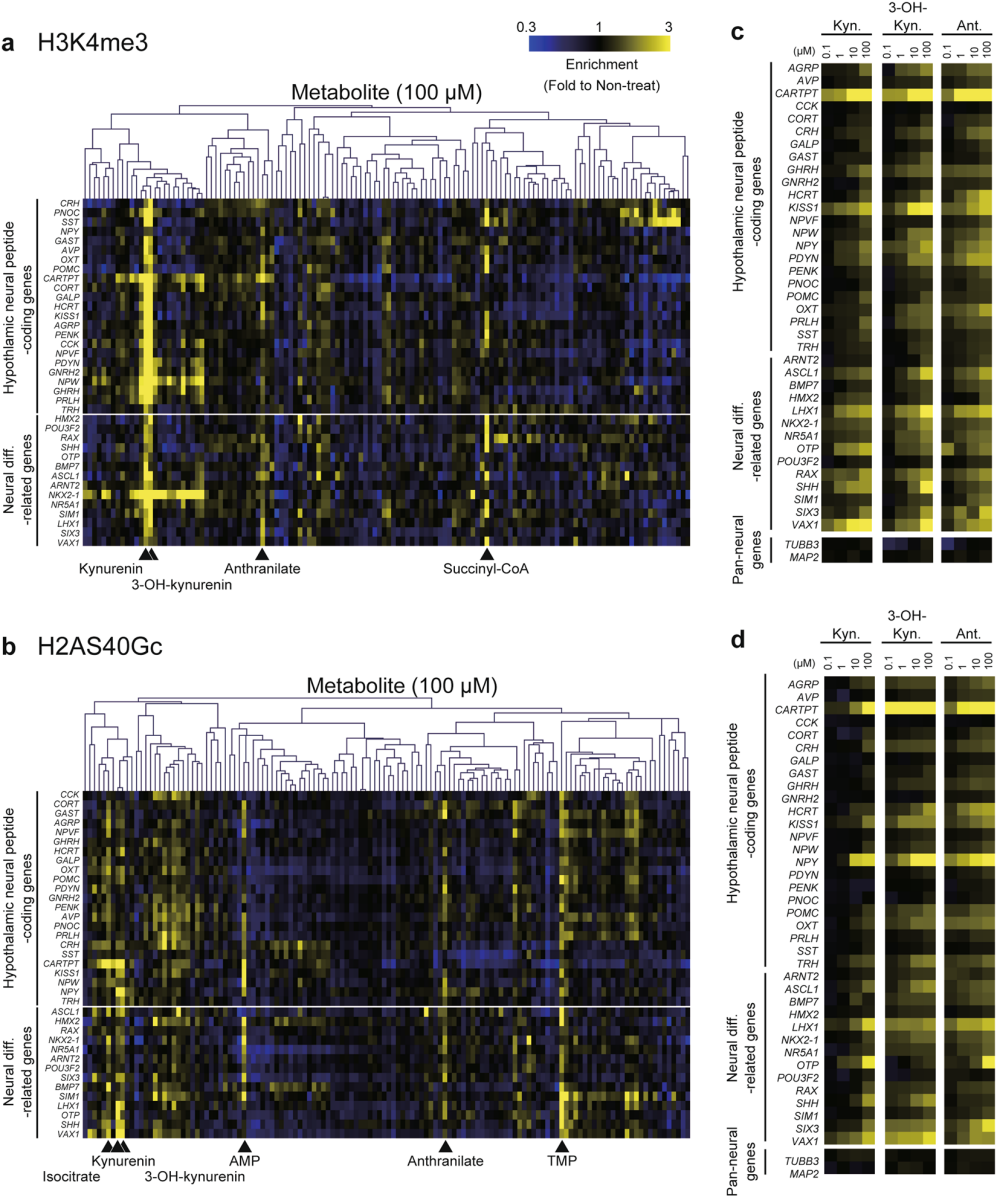
Epigenetic activation at specific loci after supplementation of kynurenine, 3-OH-kynurenine, and anthranilate. Levels of H3K4me3 (**a**) and H2AS40Gc (**b**) on hypothalamic-neural genes in cells treated with metabolites. Heatmaps show ChIP-qPCR data. Values were normalized using input data. Color scale bars indicate histone modification level of each gene in treated cells, relative to non-treated cells. Dose-dependency of H3K4me3 (**c**) and H2AS40Gc (**d**) levels after kynurenine, 3-OH-kynurenine, and anthranilate supplementation. Kyn, kynurenine. 3-OH-kyn, 3-OH-kynurenine. Ant, anthranilate.

Heatmap visualizations of ChIP-qPCR data showed that both types of histone modification increased with supplementation of kynurenine, 3-OH-kynurenine, and anthranilate at nearly every included locus (Fig. 2a,b), in a dose-dependent manner (Fig. 2c,d). The only exception was at genes encoding pan-neural markers (*TUBB3* and *MAP2*), suggesting a locus specificity to the metabolites’ effects.

**Figure 3:**
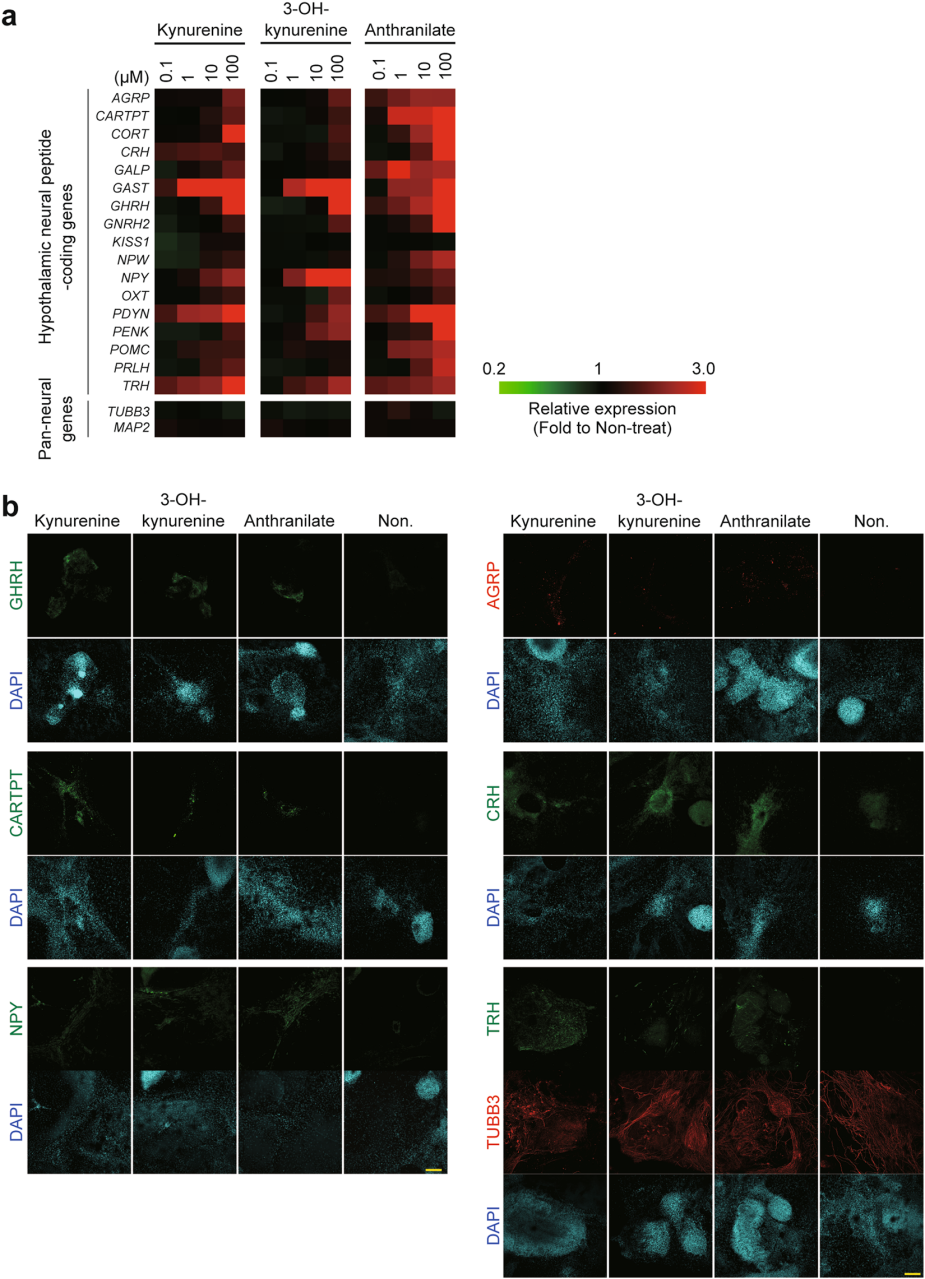
Elevated expression of genes encoding hypothalamic neuropeptides after supplementation with kynurenine, 3-OH-kynurenine, and anthranilate. (**a**) Results from RT-qPCR determining mRNA levels of neural peptide-coding genes, normalized to *ACTB* expression and visualized as a heatmap. Color scale bars indicate individual gene expression relative to expression in non-treated cells. (**b**) Immunofluorescent (IF) assays for neural peptides: GHRH, CARTPT, NPY, AGRP, CRH, and TRH. TUBB3 is a pan-neural marker. Scale bars, 200 μm.

Next, the results of RT-qPCR assays for 17 hypothalamic neuropeptide-coding genes revealed a significant, dose-dependent increase in gene expression (except of *KISS1*) in neurons supplemented with the three metabolites (Fig. 3a). Immunofluorescence assays detected more GHRH, CARTPT, NPY, AGRP, CRH, and TRH-positive cells in TUBB3-positive colonies that differentiated in the presence of kynurenine, 3-OH-kynurenine, and anthranilate than in non-treated cells (Fig. 3b).

Taken together, these results indicate that these three metabolites can change the epigenetic status at hypothalamic neural peptide-coding and neural differentiation-related loci, even though they do not directly induce epigenetic modifications.

### Kynurenine-induced epigenetic activation is necessary for long-term gene expression

**Figure 4:**
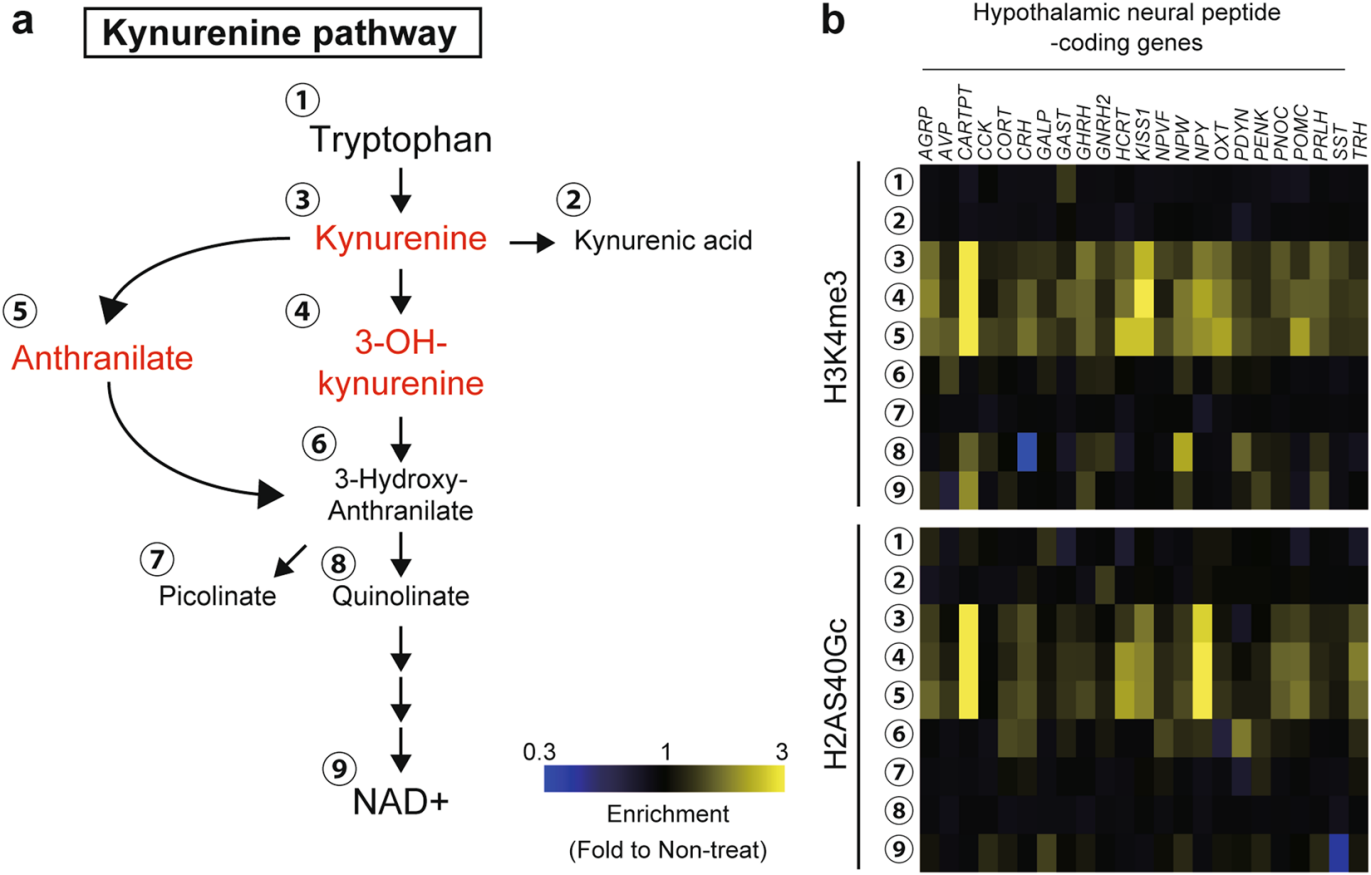
Kynurenine, 3-OH-kynurenine, and anthranilate specifically increase H3K4me3 and H2AS40Gc levels. (**a**) Schematic depicting the kynurenine pathway of tryptophan metabolism. (**b**) Effect of supplementing tryptophan and kynurenine-pathway metabolites on H3K4me3 and H2AS40Gc levels at hypothalamic neural peptide-coding loci.

Kynurenine, 3-OH-kynurenine, and anthranilate are produced from tryptophan through the kynurenine pathway (KP), which eventually produces NAD+^24,25^ (Fig. 4a). To verify whether elevated H3K4me3 and H2AS40Gc were specific to supplementation of the three metabolites, we performed another ChIP-qPCR assay of hypothalamic neural peptide-coding and differentiation-related genes. Cell cultures were supplemented with tryptophan (to trigger KP) and metabolites produced during KP. The results showed that only kynurenine, 3-OH-kynurenine, and anthranilate elevated H3K4me3 and H2AS40Gc at nearly all loci (Fig. 4b and Supplementary Fig. 1). In contrast, tryptophan and metabolites located downstream of kynurenine did not generate significant changes.

**Figure 5:**
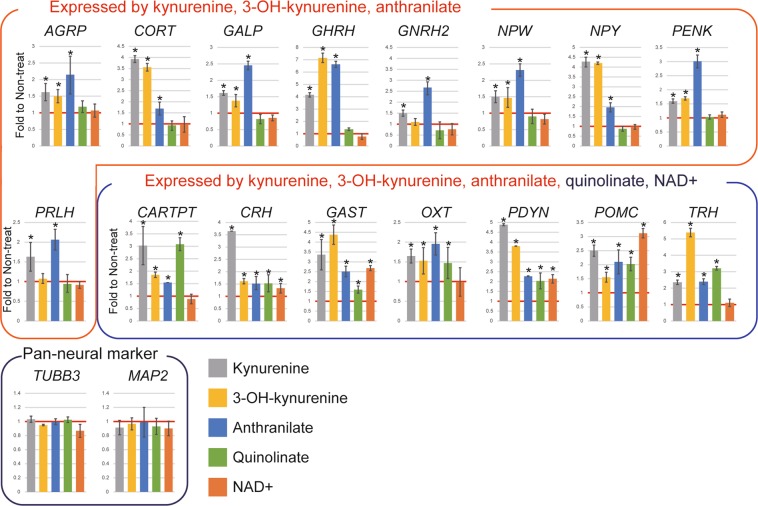
Response of gene expression to supplementing cells with quinolinate and NAD+, both located downstream of kynurenine in the kynurenine pathway. Gene expression was measured in neurons at day 24 using RT-qPCR, then normalized to *ACTB* expression. Means ± SD (n = 3). Relative values were based on the expression of non-treated cells equaling 1. *TUBB3* and *MAP2* were used as pan-neural markers. *P < 0.05.

We then performed RT-qPCR to evaluate gene expression in cells supplemented with quinolinate and NAD+, both downstream of kynurenine. Unexpectedly, this supplementation significantly upregulated the expression of some genes (*CARTPT*, *CRH*, *GAST*, *OXT*, *PDYN*, *POMC* and *TRH*) (Fig. 5).

**Figure 6:**
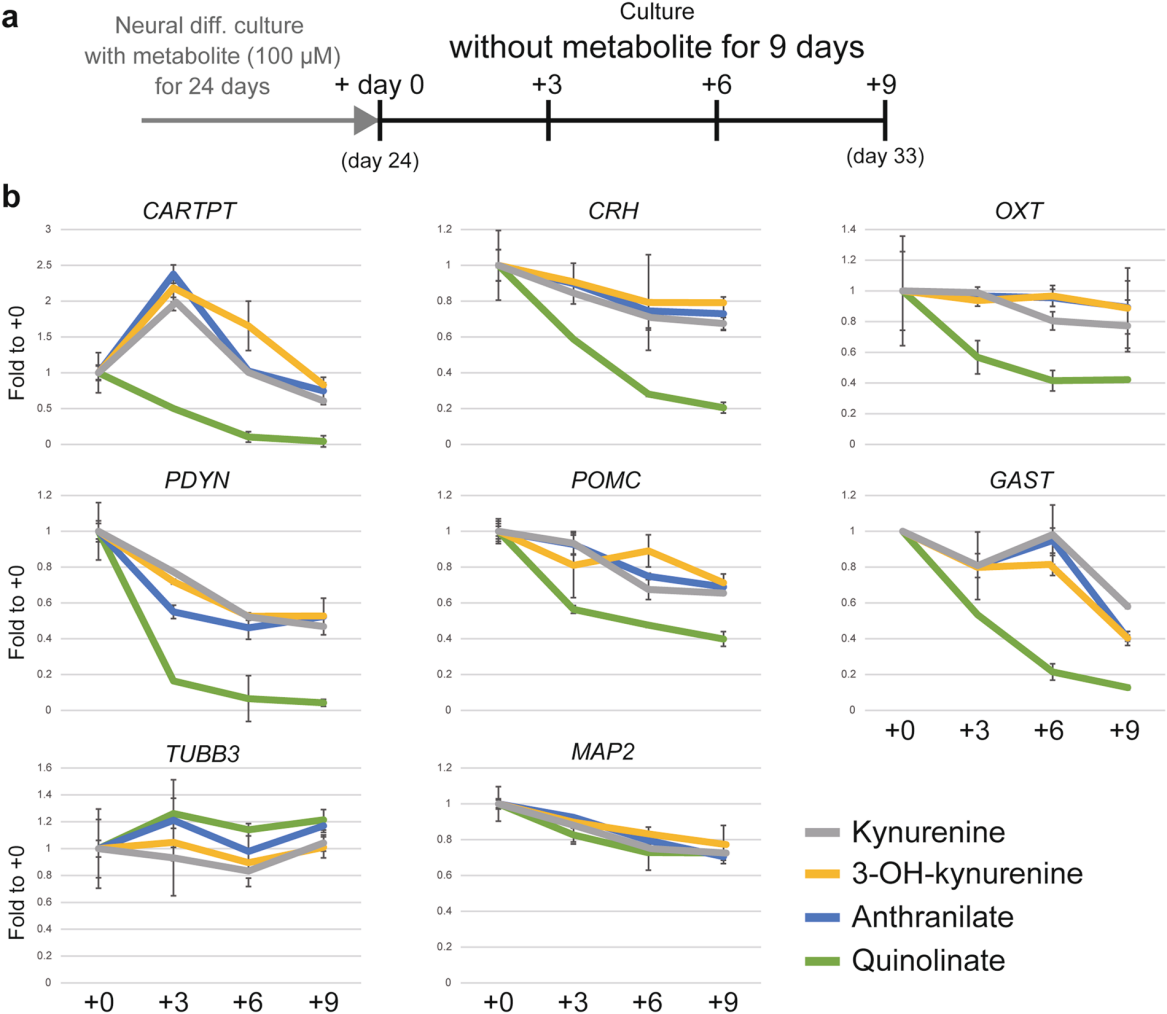
Epigenetic activation by kynurenine, 3-OH-kynurenine, and anthranilate is necessary for maintenance of expression of hypothalamic neural peptide-coding genes. (**a**) Culture protocol for analyzing gene expression in long-term-cultured cells. Kynurenine, 3-OH-kynurenine, anthranilate, and quinolinate (all 100 μM) were added on day 14. Culturing continued until day 24, followed by 9 days of culture without metabolites. (**b**) Neural peptide-coding gene expression, evaluated with RT-qPCR, in neurons cultured without metabolites for 9 days. Means ± SD (n = 3). *TUBB3* and *MAP2* were used as pan-neural markers.

Epigenetic mechanisms support stable gene activation, thus allowing for specific functions in distinct cell types^1,2^. We cultured cells for 9 days without metabolites to clarify whether epigenetic changes did indeed result in gene upregulation (Fig. 6a). During this period, six genes (*CARTPT*, *CRH*, *OXT*, *PDYN*, *POMC*, and *GAST*) maintained the same expression in cells supplemented with kynurenine, 3-OH-kynurenine, and anthranilate (Fig. 6b). However, in quinolinate-supplemented cells, the expression of these genes dropped quickly. The pan-neural marker genes *TUBB3* and *MAP2* did not exhibit these differences between culture conditions.

Collectively, these data show that the three metabolites (kynurenine, 3-OH-kynurenine, and anthranilate) were necessary for epigenetic activation (H3K4me3 and H2AS40Gc). Moreover, these epigenetic changes are necessary for the maintenance of stable gene expression.

### Kynurenine, 3-OH-kynurenine, and anthranilate did not affect the expression of genes involved in histone modification and NAD production

**Figure 7:**
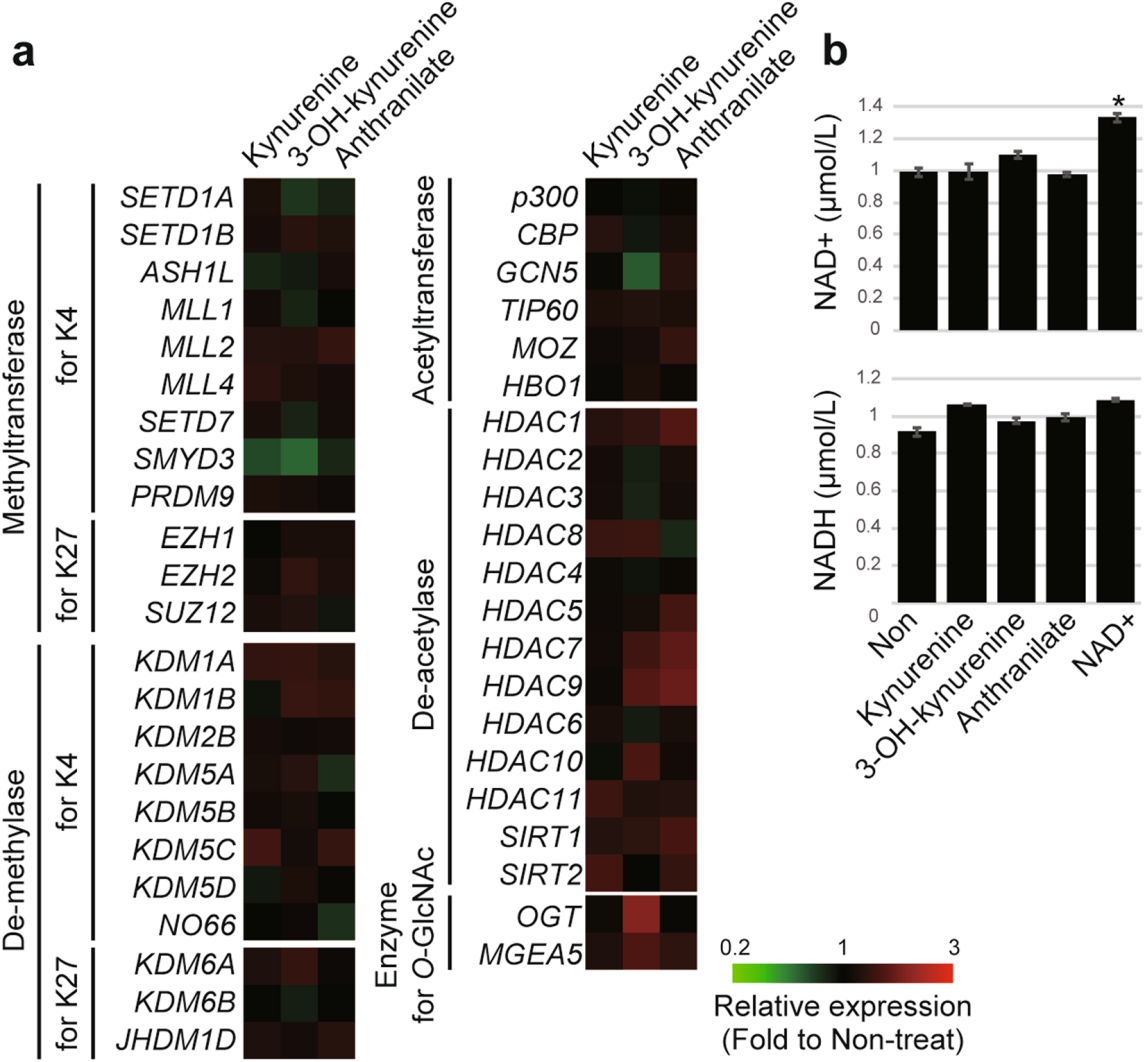
Supplementation of kynurenine, 3-OH-kynurenine, and anthranilate did not alter expression of histone-modification-enzyme-coding genes or NAD concentration. (**a**) Expression of histone-modifier-encoding genes was evaluated using RT-qPCR, normalized to *ACTB*, and visualized as a heatmap. Color scale bars indicate relative expression to non-treated cells. (**b**) Concentrations of intracellular NAD+ and NADH in neurons supplemented with kynurenine, 3-OH-kynurenine, anthranilate, or NAD+. Means ± SD (n = 3). *P < 0.05.

To understand the mechanisms underlying kynurenine-induced epigenetic activation, we analyzed the expression of genes encoding histone modification enzymes in neurons supplemented with kynurenine, 3-OH-kynurenine, and anthranilate. We also measured the intracellular NAD+ and NADH concentrations in these cells. We did not find significant metabolite-related changes in the expression of genes coding enzymes for H3K4me3 (17 genes) or *O*-GlcNAcylation (two genes) (Fig. 7a). We also examined the genes associated with repressed histone modification H3K27me3 (six genes) and histone acetylation (19 genes) because multiple layers of epigenetic regulatory controls involving histone modifications are involved in the establishment, maintenance, and heritability of gene expression^26,27^. We also looked for and did not find any metabolite-induced changes in these genes.

NAD+ is an activator of sirtuins, a group of molecules that function as histone de-acetylases^28,29^. Therefore, cellular NAD+ concentration and NAD+/NADH balance are important for histone acetylation, indirectly leading to kynurenine-dependent epigenetic activation. We measured NAD+ and NADH concentrations in cells supplemented with kynurenine, 3-OH-kynurenine, and anthranilate, using NAD-supplemented cells as a positive control. We found that intracellular NAD+ and NADH concentrations did not significantly change in cells treated with the three metabolites (Fig. 7b).

## Discussion

We successfully demonstrated that metabolites kynurenine, 3-OH-kynurenine, and anthranilate activate histone modifications at loci encoding hypothalamic neural peptides and differentiation-related factors. Further, these epigenetic modifications were associated with gene expression and necessary for the maintenance of stable gene expression. Previous reports examining the link between epigenetics and nutrition have tended to focus on one-carbon metabolism, glycolysis, and acyl-CoA metabolism, which directly provides SAM and acetyl-CoA for protein methylation and acetylation, respectively^30,31^. Our study added to these findings by showing that metabolites not directly involved in epigenetic modifications can also influence epigenetic status.

In this study, we used a ChIP-qPCR assay to estimate locus-specific epigenetic alterations. We chose not to use whole-measurement methods such as western blots. Although previous studies indicated that intracellular concentrations of epigenetic factors (e.g., acetyl-CoA and SAM) affected global modification levels^5,32^, this occurred in a locus-specific manner^33,34^. Our previous study also indicated that ManNAc activated only *HCRT* and not other loci coding hypothalamic neural peptides^15,16^. Our decision to use locus-specific methods allowed us to better identify the link between individual metabolites (kynurenine, 3-OH-kynurenine, anthranilate) and a given locus. By changing the gene set and cell type in each experiment, our method could find metabolites that alter epigenetic status in a cell-type- and gene-specific manner.

The essential amino acid tryptophan is either used for protein synthesis or metabolized into bioactive molecules via the KP or serotonin pathway; these processes account for over 95% of dietary tryptophan^24,25^. Most KP metabolites are neuroactive, with essential roles in regulating *N*-methyl-D-aspartate (NMDA) receptor function and free-radical production^35^. Since NMDA receptor-mediated excitotoxicity and excessive free-radical production are involved in neurodegenerative disorders, including Huntington’s, Parkinson’s, and Alzheimer’s diseases, preclinical studies are investigating pharmacological modulation of this pathway^36,37^. Although research has shown that kynurenine and 3-OH-kynurenine concentrations are elevated in the cerebrospinal fluid of patients with neurological diseases^38,39^, the link between altered metabolite levels and disease development remains unclear. Given that kynurenine can alter epigenetic status in addition to regulating NMDA receptor function, our findings provide new insight on how KP metabolites could cause some neurological disorders. In the present study, we fixed the metabolite supplementation time at 10 days. To determine the link between the epigenetic function of KP metabolites and disease development, it may also be important to understand the timing and duration of metabolite exposure needed for altering epigenetic status. Future time-course experiments on neurons can help validate this hypothesis.

Here, we mainly focused on the effects of kynurenine, 3-OH-kynurenine, and anthranilate because they elevated both H3K4me3 and H2AS40Gc levels. However, we found that supplementation with succinyl-CoA increased H3K4me3 levels, while supplementation with isocitrate, AMP, and TMP increased H2AS40Gc levels. Succinyl-CoA, isocitrate, and AMP have been linked to epigenetic regulation. Instrumental in tumor development, succinyl-CoA is involved in histone succinylation through the action of lysine acetyltransferase 2A (KAT2A)^40^. Isocitrate is a precursor of α-KG, a metabolic intermediate that acts as a cofactor for several chromatin-modifying enzymes, including histone demethylases and TET enzymes^41^. Lastly, AMP triggers AMP-activated kinase (AMPK), a master regulator of energy homeostasis, as well as a key mediator of adaptation and cell survival through phosphorylation of histones, DNA methyltransferases, and histone modifiers^42,43^. All of these metabolites might have influenced H3K4me3 or H2AS40Gc levels, however, further research is needed to clarify the exact mechanism through which the metabolites influence H3K4me3 or H2AS40Gc.

Here, we investigated whether kynurenine supplementation would indirectly affect expression of epigenetic factors and intracellular NAD+ concentration. However, we could not determine the mechanisms from our results. Previous reports suggest that kynurenine binds to the transcription factor aryl hydrocarbon receptor (AhR), inducing its translocation from the cytoplasm to the nucleus^44^. AhR is implicated in multiple cellular processes, including embryogenesis, tumorigenesis, and inflammation^45,46^. Moreover, AhR regulates the expression of genes encoding epigenetic factors such as *HDAC8*^47^. However, supplementation with kynurenine, 3-OH-kynurenine, and anthranilate did not affect HDAC gene expression in this study. Currently, we lack a clear understanding of AhR as an epigenetic factor, nor are data available on the binding potency of 3-OH-kynurenine and anthranilate to AhR. We recommend further studies focusing on AhR to determine whether it is a key player in epigenetic activation through supplementation of kynurenine, 3-OH-kynurenine, and anthranilate.

Key questions for the future include delineating the mechanisms underlying activation of epigenetic status by kynurenine at specific loci and regulation of nuclear events. Metabolic enzymes may adjust gene transcription in response to changes in metabolic state, although few studies have provided empirical evidence of this phenomenon. Fumarase, an enzyme normally associated with oxidative metabolism in the mitochondria, is localized in the nucleus through interacting with histone variant H2AZ, and regulates both histone methylation and DNA double-strand break repair^48^. An enzyme involved in SAM production, MAT2A also localizes to the nucleus and is associated with transcriptional repression of *COX2*, a process mediated by H3K9 methylation through the action of methyltransferase SETDB1^34^. Although traditionally located in the mitochondria, the pyruvate dehydrogenase complex can move to the nucleus, where it generates high acetyl-CoA concentrations to fuel histone acetylation, required for gene expression during the S phase^49^. According to the Human Protein Atlas (https://www.proteinatlas.org/)^50^, enzymes involved in kynurenine, 3-OH-kynurenine, and anthranilate metabolism (e.g., KYNU and KYAT1) are localized to the cytoplasm and nucleoplasm (Supplementary Fig. 2). Experimental findings confirm that tryptophan degradation during KP occurs in the cytoplasm^24,25^. These data suggest that nuclear localization of KYNU or KYAT can dramatically affect chromatin structure by change in metabolism, resulting in epigenetic changes and regulation of gene expression. KYNU and KYAT are of particular interest because as of now, there are no reports on the relationship between kynurenine metabolism and epigenetic systems such as H3K4me3 and H2AS40Gc; therefore, the mechanisms underlying kynurenine-dependent epigenetic alterations are unknown. However, nucleus-localized KYNU and KYAT could be key factors in understanding those mechanisms. To clarify the mechanisms, future studies should analyze the physiological functions of these epigenetic regulation-related enzymes and their partner proteins.

In conclusion, the present study, which screened metabolites capable of altering the epigenetic status at specific loci, indicated that kynurenine, 3-OH-kynurenine, and anthranilate function to activate epigenetic status involved in induction/maintenance of gene expression. Our results will provide a new insight for controlling cell fate and disease progression by exploiting the epigenetic modulation ability of these metabolites.

## Materials and Methods

### Reagents

Unless otherwise noted, reagents were purchased from Wako Pure Chemical (Japan). Metabolites used in this study are listed in Supplementary Table 1. All primers were prepared by Sigma or FASMAC; their sequences are shown in Supplementary Table S2. Antibodies are listed in Supplementary Table S3.

### Neural differentiation from hiPSCs

The hiPSC line 201B7 was provided by RIKEN BRC, through the National Bio-Resource Project of the MEXT, Japan^51^. Cells were maintained on layers of mitomycin C-treated STO/Neo resistant/LIF (SNL) feeder cells in ReproStem medium (ReproCELL, Japan) supplemented with 5 ng/mL bFGF. Neural differentiation using SDIA methods was performed as described in the previous reports^23^. Briefly, the hiPSCs (1.7 × 10^3^ cells/cm^2^) were cultured on PA6 feeder cells (RIKEN BRC, Japan) in G-MEM supplemented with 10% serum replacement, 0.1 mM NEAA, and 0.1 mM β-mercaptoethanol. The culture medium was changed on days 1 and 4, then every 3 d thereafter. We separately added selected metabolites (100 μM each) to each culture medium beginning on day 14 and ending on day 24.

### Chromatin immunoprecipitation (ChIP)

The assay was performed with 1 × 10^6^ cells per assay using the ChIP-IT Express Enzymatic Kit (Active Motif, USA), following the manufacturer’s protocol. Briefly, fixed cells were lysed and subjected to an enzymatic shearing cocktail for 10 min to cut chromatin. After immunoprecipitation, DNA was recovered using an elution buffer (10% SDS, 300 mM NaCl, 10 mM Tris-HCl, and 5 mM EDTA; pH 8.0) at 65 °C overnight and then collected using the ChIP DNA Clean and Concentrator Kit (Zymo Research, USA). The 10% input (DNA without IP) and normal mouse IgGs (mIgG) were used as the positive and negative controls, respectively.

### mRNA extraction and cDNA synthesis

Total RNA was isolated from cells using the Direct-zol RNA MiniPrep Kit (Zymo Research) following the manufacturer’s protocol. First-strand cDNA was synthesized from 500 ng of RNA using ReverTra Ace qPCR RT Master Mix with gDNA Remover (TOYOBO, Japan).

### Quantitative PCR (qPCR)

A high-throughput gene expression platform based on microfluidic dynamic arrays (Fluidigm, USA) was used to perform ChIP-qPCR and RT-qPCR. The resultant DNA was pre-amplified using TaqMan PreAmp MasterMix (Applied Biosystems, USA) following the manufacturer’s protocol. Following pre-amplification, samples were diluted 1:5 in TE buffer (pH 8.0). BioMark 48 × 48 arrays were prepared following the manufacturer’s protocol. After the IFC controller loaded assays and samples into the chip, PCR was performed under the following conditions: 50 °C for 2 min and 95 °C for 10 min, followed by 40 cycles of 95 °C for 15 s and 60 °C for 60 s. Data were processed in BioMark Real-time PCR Analysis software (Fluidigm), through automatic threshold setting (same value for all assays) and linear baseline correction. Data from ChIP-qPCR and RT-qPCR were normalized to input DNA and *ACTB* expression, respectively.

Mean qPCR data from three independent reactions were visualized using PCA and heatmaps in the Galaxy browser (www.galaxy.psu.edu) and MeV^52^, respectively.

### Immunofluorescent staining

Cells cultured in four-well dishes were fixed using 4% paraformaldehyde and permeabilized with 0.2% Triton X-100. They were blocked using 5% bovine serum albumin (BSA)/0.1% Tween 20/phosphate-buffered saline for 1 h at 24 °C and incubated with primary antibody overnight at 4 °C. After adding the secondary antibody, incubation continued for another hour at 24 °C. Nuclei were stained using DAPI (1 μg/mL, Dojindo, Japan). Fluorescence images were acquired with a confocal laser scanning microscope (LSM700, ZEISS, Germany).

### Measurement of NAD+ and NADH

Cells were cultured on six-well dishes. Kynurenine, 3-OH-kynurenine, anthranilate, and NAD+ were added at 100 μM 1 h before measurements. NAD + and NADH concentrations were determined with the NAD/NADH Assay Kit-WST (Dojindo), following the manufacturer’s protocol.

### Statistical analyses

Student’s *t*-tests were performed to compare qPCR and NAD+/NADH assay data. Significance was set at *P* < 0.05. All experiments were repeated at least three times, with similar results obtained in each case. The data shown are representative of repeated experiments.

## Supplementary information

Supplementary information

## Data Availability

All data generated during this study are included in the published article and Supplementary Information Files.
