# Supplementary material for: Kynurenine, 3-OH-kynurenine, and anthranilate are nutrient metabolites that alter H3K4 trimethylation and H2AS40 O-GlcNAcylation at hypothalamus-related loci
Source: Sci Rep. 2019 Dec 24;9:19768. doi: 10.1038/s41598-019-56341-x (PMC6930210; doi:10.1038/s41598-019-56341-x)
Supplement: Supplementary file 1 — Supplementary information [file 41598_2019_56341_MOESM1_ESM.docx]

**Supplementary information**

**Kynurenine, 3-OH-kynurenine, and anthranilate are nutrient metabolites that alter H3K4 trimethylation and H2AS40 *O*-GlcNAcylation at hypothalamus-related loci**

Koji Hayakawa^1, 2^ *, Kenta Nishitani^2^ and Satoshi Tanaka^2^

^1^, Department of Toxicology, Faculty of Veterinary Medicine, Okayama University of Science, Imabari-shi, Ehime, Japan

^2^, Laboratory of Cellular Biochemistry, Department of Animal Resource Sciences /Veterinary Medical Sciences, The University of Tokyo, Tokyo, Japan

* To whom correspondence should be addressed: Koji Hayakawa. Department of Toxicology, Faculty of Veterinary Medicine, Okayama University of Science, Imabari-shi, Ehime, Japan. Tel: +81-898-52-9130, E-mail: k-hayakawa@vet.ous.ac.jp.


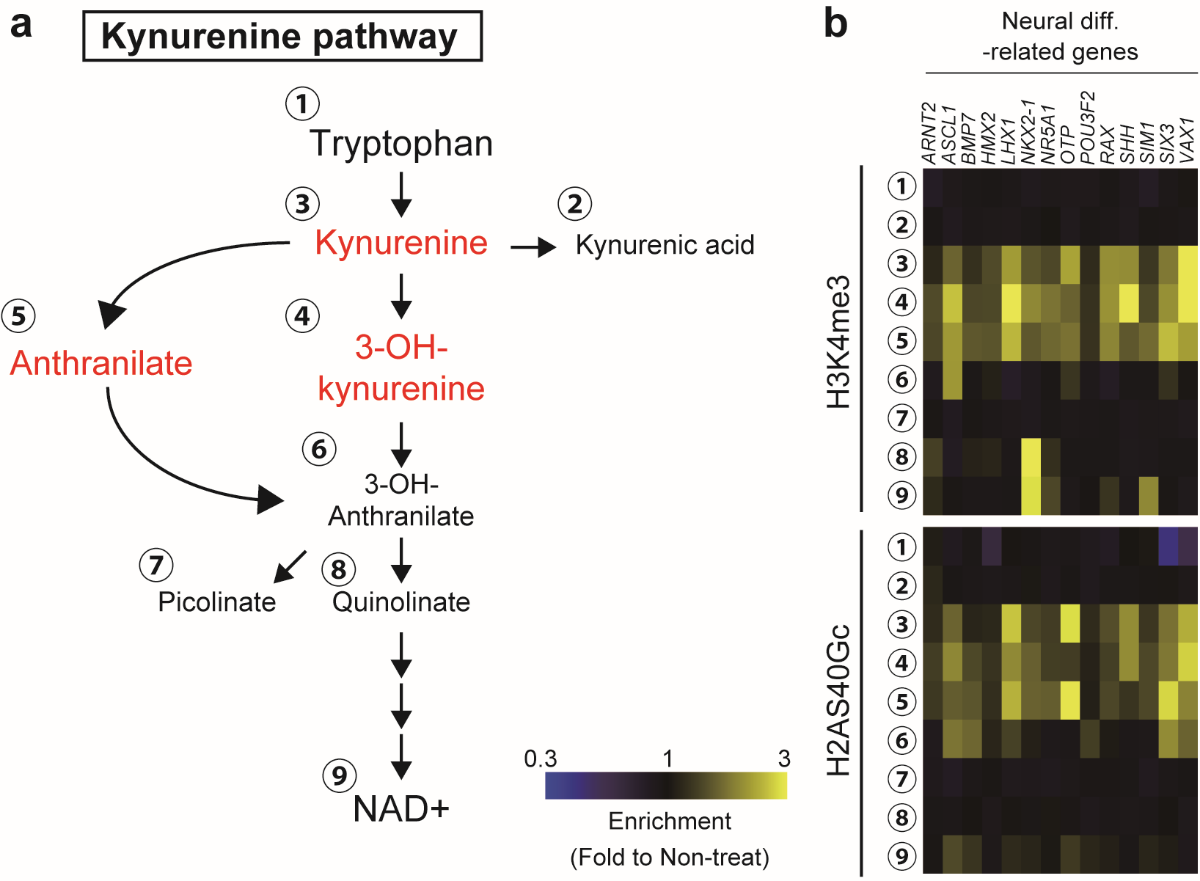


**Supplementary Figure 1 Effect of supplementing tryptophan and kynurenine-pathway metabolites on H3K4me3 and H2AS40Gc levels at hypothalamic neural differentiation-related loci.**

(**a**) Schematic depicting the kynurenine pathway of tryptophan metabolism. (**b**) Heatmaps show ChIP-qPCR data. Values were normalized using input data. Color scale bars indicate histone modification level of each gene in treated cells, relative to non-treated cells.


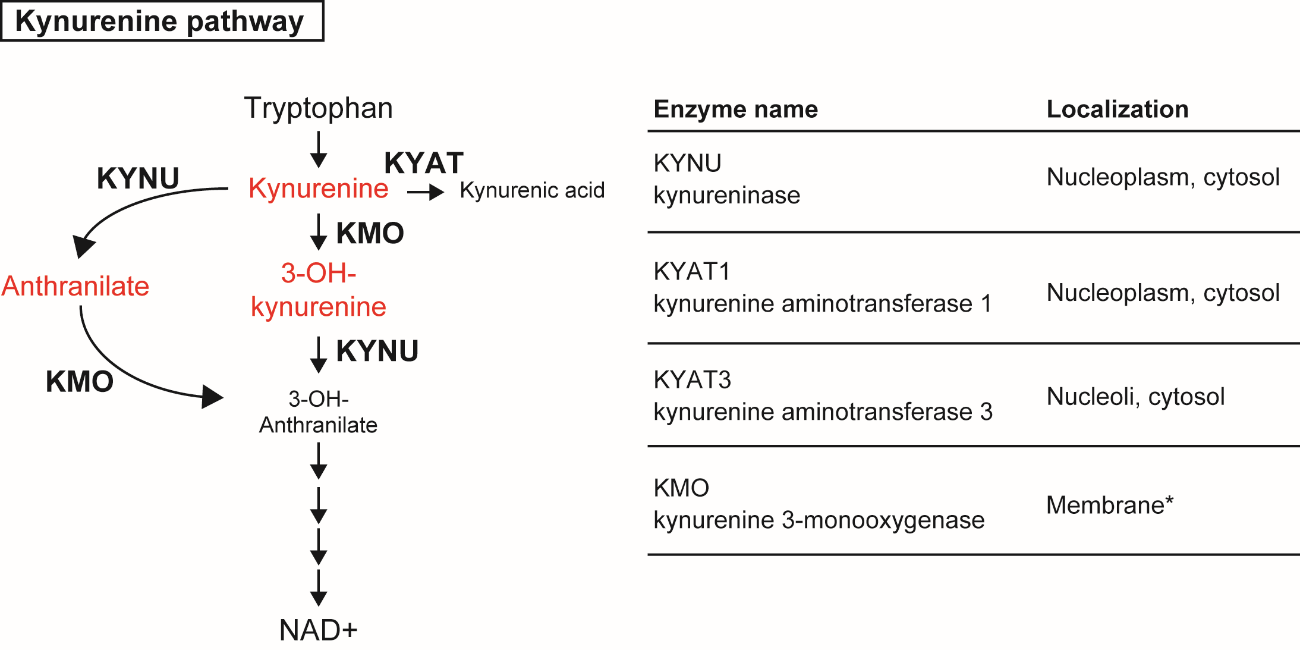


**Supplementary Figure 2 Intracellular localization of enzymes for kynurenine, 3-OH-kynurenine and anthranilate.**

According to the data of HUMAN PROTEIN ATLAS (https://www.proteinatlas.org/), Intracellular localization of KYNU, KYAT1, KYAT3 and KMO was listed. *, predicted localization.

**Supplementary Table 1**

| Common Name | CAS no. | Company | Cat. no. | Class | Direct Parent |
| --- | --- | --- | --- | --- | --- |
| NAD | 53-84-9 | SIGMA | N3014-100MG | Nucleosides, nucleotides, and analogues | (5'->5')-dinucleotides |
| S-5’Adenosyl-L- homocysteine | 979-92-0 | SIGMA | A9384-25MG |  | 5'-deoxy-5'-thionucleosides |
| dGMP | 33430-61-4 | SIGMA | 852228-1G |  | Purine 2'-deoxyribonucleoside diphosphates |
| dADP | 72003-83-9 | SIGMA | D6000-25MG |  |  |
| Inosine | 58-63-9 | TCI Chemicals | I0037 |  | Purine nucleosides |
| Guanosine diphosphate mannose | 103301-73-1 | SIGMA | G5131-10MG |  | Purine nucleotide sugars |
| ADP-glucose | 102129-65-7 | SIGMA | A0627-10MG |  |  |
| GDP-glucose | 103301-72-0 | SIGMA | G7502-10MG |  |  |
| GDP-L-fucose | 15839-70-0 | SIGMA | G4401-1MG |  |  |
| Dephospho-CoA | 3633-59-8 | SIGMA | D3385-5MG |  | Purine ribonucleoside diphosphates |
| Guanosine diphosphate | 43139-22-6 | SIGMA | G7127-25MG |  |  |
| ADP | 16178-48-6 | TCI Chemicals | A0626 (100 mg) |  |  |
| Inosinic acid | 352195-40-5 | TCI Chemicals | I0036 (1 g) |  | Purine ribonucleoside monophosphates |
| Adenylsuccinic acid | 19046-78-7 | SIGMA | A5028-25MG |  |  |
| AMP | 61-19-8 | TCI Chemicals | A0158 (1 g) |  |  |
| dTDP | 108322-12-9 | SIGMA | T9375-5MG |  | Pyrimidine 2'-deoxyribonucleoside diphosphates |
| dCMP | 1032-65-1 | TCI Chemicals | D3673 (100 mg) |  | Pyrimidine 2'-deoxyribonucleoside monophosphates |
| dUMP | 42155-08-8 | SIGMA | D3876-100MG |  |  |
| Uridine diphosphate-N-acetylglucosamine | 91183-98-1 | SIGMA | U4375-25MG |  | Pyrimidine nucleotide sugars |
| UDP-N-acetyl-alpha-D-galactosamine | 108320-87-2 | SIGMA | U5252-5MG |  |  |
| Uridine diphosphate glucose | 28053-08-9 | SIGMA | U4625-10MG |  |  |
| Uridine diphosphate glucuronic acid | 63700-19-6 | SIGMA | U6751-25MG |  |  |
| Uridine diphosphategalactose | 137868-52-1 | SIGMA | U4500-10MG |  |  |
| Uridine 5'-diphosphate | 27821-45-0 | SIGMA | 94330-100MG |  | Pyrimidine ribonucleoside diphosphates |
| CDP | 34393-59-4 | TCI Chemicals | C0523 |  |  |
| Uridine 5'-monophosphate | 3387-36-8 | TCI Chemicals | U0021 |  | Pyrimidine ribonucleoside monophosphates |
| 5-Thymidylic acid | 33430-62-5 | SIGMA | T7004-100MG | Pyrimidine nucleotides | Pyrimidine 2'-deoxyribonucleoside monophosphates |
| S-adenosyl-L-methioninate | 86867-01-8 | SIGMA | A7007-100MG | 5'-deoxyribonucleosides | 5'-deoxy-5'-thionucleosides |
| DL-O-Phosphoserine | 17885-08-4 | SIGMA | 79710-10G | Organic acids and derivatives | Alpha amino acids |
| DL-Homocysteine | 454-29-5 | SIGMA | H4628-1G |  |  |
| DL-2-Aminoadipic acid | 542-32-5 | SIGMA | A0637-1G |  |  |
| Creatine | 6020-87-7 | SIGMA | C3630-100G |  | Alpha amino acids and derivatives |
| L-Dihydroorotic acid | 5988-19-2 | SIGMA | D7128-500MG |  |  |
| Ureidosuccinic acid | 923-37-5 | TCI Chemicals | C0029 |  | Aspartic acid and derivatives |
| 3-Aminoisobutanoic acid | 144-90-1 | SIGMA | 217794-1G |  | Beta amino acids and derivatives |
| Hydroxypyruvic acid | 1113-60-6 | SIGMA | 06372-5MG-F |  | Beta hydroxy acids and derivatives |
| Chorismate | 617-12-9 | SIGMA | C1761-5MG |  | Dicarboxylic acids and derivatives |
| Gamma-Aminobutyric acid | 56-12-2 | Wako | 018-02442 |  | Gamma amino acids and derivatives |
| Saccharopine | 997-68-2 | SIGMA | S1634-10MG |  | Glutamic acid and derivatives |
| L-Homoserine | 672-15-1 | SIGMA | H6515-10MG |  | L-alpha-amino acids |
| Citrulline | 372-75-8 | SIGMA | C7629-1G |  |  |
| L-Cystathionine | 56-88-2 | SIGMA | C7505-10MG |  | L-cysteine-S-conjugates |
| Oxoadipic acid | 3184-35-8 | SIGMA | 75447-100MG |  | Medium-chain keto acids and derivatives |
| Gamma-Glutamylcysteine | 636-58-8 | SIGMA | G0903-25MG |  | N-acyl-alpha amino acids |
| N-Acetylserine | 97-14-3 | SIGMA | A2638-1G |  | N-acyl-L-alpha-amino acids |
| Phosphoenolpyruvic acid | 5541-93-5 | SIGMA | P7002-100MG |  | Phosphate esters |
| Oxaloacetic acid | 328-42-7 | SIGMA | O4126-1G |  | Short-chain keto acids and derivatives |
| Hypotaurine | 300-84-5 | SIGMA | H1384-100MG |  | Sulfinic acids |
| Isocitric acid | 1637-73-6 | SIGMA | I1252-1G |  | Tricarboxylic acids and derivatives |
| Ureidopropionic acid | 462-88-4 | SIGMA | 94295-1G |  | Ureas |
| Dihydrothymine | 696-04-8 | SIGMA | S412295-50MG |  | Ureides |
| N-Acetyl-D-Glucosamine 6-Phosphate | 102029-88-9 | SIGMA | A4394-25MG | Organic oxygen compounds | Acyl amino sugars |
| N-Acetylmannosamine | 7772-94-3 | Sanyo fine |  |  |  |
| L-Kynurenine | 2922-83-0 | SIGMA | K8625-25MG |  | Alkyl-phenylketones |
| Hydroxykynurenine | 2147-61-7 | SIGMA | H1771-25MG |  |  |
| Phospho-hydroxypyruvic acid | 3913-50-6 | SIGMA | 02711-10MG |  | Glycerone phosphates |
| Glucose 6-phosphate | 56-73-5 | SIGMA | G7375-1G |  | Hexose phosphates |
| Fructose 6-phosphate | 26177-86-6 | SIGMA | F3627-10MG |  |  |
| Glucosamine 6-phosphate | 70442-23-8 | SIGMA | G4878-100MG |  |  |
| Mannose 6-phosphate | 70442-25-0 | SIGMA | M3655-100MG |  |  |
| Fructose 1,6-bisphosphate | 38099-82-0 | SIGMA | F6803-10MG |  |  |
| D-Mannose 1-phosphate | 27251-84-9 | SIGMA | M1755-10MG |  | Monosaccharide phosphates |
| Glucose 1-phosphate | 56401-20-8 | SIGMA | G7000-1G |  |  |
| N-Acetyl-glucosamine 1-phosphate | 31281-59-1 | SIGMA | A2142-5MG |  | N-acyl-alpha-hexosamines |
| N-Acetylneuraminic acid | 131-48-6 | TCI Chemicals | A0639 |  | N-acylneuraminic acids |
| dCDP | 151151-32-5 | SIGMA | D7250-5MG |  | Organic pyrophosphates |
| 3-Phosphoglyceric acid | 80731-10-8 | SIGMA | P8877-10MG |  | Sugar acids and derivatives |
| 2-Phosphoglyceric acid | 83418-48-8 | SIGMA | 79470-50MG |  |  |
| L-Histidinol | 1596-64-1 | SIGMA | H6647-10MG |  | Aralkylamines |
| Spermine | 71-44-3 | SIGMA | S4264-1G |  | Dialkylamines |
| Spermidine | 124-20-9 | SIGMA | S0266-1G |  |  |
| Betaine aldehyde | 7758-31-8 | SIGMA | B3650-2MG |  | Tetraalkylammonium salts |
| Shikimic acid 3-phosphate | 143393-03-7 | SIGMA | S0702-1MG | Organic phosphoric acids and derivatives | Monoalkyl phosphates |
| Taurine | 107-35-7 | wako | 201-00112 | Organic sulfonic acids and derivatives | Organosulfonic acids |
| N-Acetylserotonin | 1210-83-9 | SIGMA | A1824-100MG | Organoheterocyclic compounds | Hydroxyindoles |
| Thymine | 65-71-4 | Wako | 205-01391 |  | Hydroxypyrimidines |
| Indole | 120-72-9 | SIGMA | I3408-25G |  | Indoles |
| L-Tryptophan | 73-22-3 | Wako | 038-23581 |  | Indolyl carboxylic acids and derivatives |
| Quinolinic acid | 89-00-9 | SIGMA | P63204-25G |  | Pyridinecarboxylic acids |
| Dihydrouracil | 504-07-4 | SIGMA | D7628-5G |  | Pyrimidones |
| Kynurenic acid | 492-27-3 | TCI Chemicals | H0303 |  | Quinoline carboxylic acids |
| 5-Hydroxy-L-tryptophan | 4350-09-8 | SIGMA | H9772-100MG |  | Serotonins |
| Tryptamine | 61-54-1 | SIGMA | 193747-10G |  | Tryptamines and derivatives |
| Xanthine | 69-89-6 | Wako | 241-00013 |  | Xanthines |
| L-Carnitine | 541-15-1 | Wako | 325-64802 | Organonitrogen compounds | Carnitines |
| Putrescine | 333-93-7 | SIGMA | P5780-5G |  | Monoalkylamines |
| Sarcosine | 107-97-1 | Wako | 198-07672 | Carboxylic acids and derivatives | Alpha amino acids |
| Dimethylglycine | 2491-06-7 | Wako | 352-00391 |  |  |
| Betaine | 107-43-7 | Wako | 023-10862 |  |  |
| Pantothenic acid | 137-08-6 | SIGMA | P5155-100G |  | Beta amino acids and derivatives |
| Beta-Alanine | 107-95-9 | TCI Chemicals | A0180 |  |  |
| O-succinyl-L-homoserine | 1492-23-5 | SIGMA | S7129-25MG |  | D-alpha-amino acids |
| Succinic acid | 6106-21-4 | SIGMA | S5047-100G |  | Dicarboxylic acids and derivatives |
| L-Glutamic acid | 56-86-0 | Wako | 070-00502 |  | Glutamic acid and derivatives |
| Citric acid | 6132-04-3 | Wako | 204-16675 |  | Tricarboxylic acids and derivatives |
| Oxoglutaric acid | 22202-68-2 | SIGMA | K1875-1G |  | Gamma-keto acids and derivatives |
| Acetoacetic acid | 3483-11-2 | SIGMA | A8509-10MG |  | Short-chain keto acids and derivatives |
| 2-Ketobutyric acid | 2013-26-5 | SIGMA | K0875-5G |  |  |
| Glutaryl-CoA | 103192-48-9 | SIGMA | G9510-5MG | Lipids and lipid-like molecules | 2,3,4-saturated fatty acyl CoAs |
| Acetoacetyl-CoA | 102029-52-7 | MP Biomedicals | 150224 |  | 3-oxo-acyl CoAs |
| Succinyl-CoA | 108347-97-3 | SIGMA | S1129-5MG |  | Acyl CoAs |
| Isobutyryl-CoA | 103404-95-1 | SIGMA | I0383-5MG |  |  |
| Methylmalonyl-CoA | 104809-02-1 | SIGMA | M1762-1MG |  |  |
| Isovaleryl-CoA | 6244-91-3 | SIGMA | I9381-10MG |  |  |
| 2-Isopropylmalic acid | 3237-44-3 | SIGMA | 333115-100MG |  | Hydroxy fatty acids |
| 3-Isopropylmalate | 16048-89-8 | SIGMA | 02339-100MG |  |  |
| Orotic acid | 50887-69-9 | TCI Chemicals | O0065 | Diazines | Pyrimidinecarboxylic acids |
| Uracil | 66-22-8 | TCI Chemicals | U0013 |  | Pyrimidones |
| Hypoxanthine | 68-94-0 | Wako | 080-03401 | Imidazopyrimidines | Hypoxanthines |
| Uric acid | 69-93-2 | Wako | 216-00222 |  | Xanthines |
| Melatonin | 73-31-4 | SIGMA | M5250-250MG | Indoles and derivatives | 3-alkylindoles |
| Indoleacetic acid | 87-51-4 | SIGMA | I2886-5G |  | Indole-3-acetic acid derivatives |
| Homogentisic acid | 451-13-8 | SIGMA | H0751-100MG | Benzenoids | 2(hydroxyphenyl)acetic acids |
| 3-Hydroxyanthranilic acid | 548-93-6 | TCI Chemicals | A0316 |  | Hydroxybenzoic acid derivatives |
| Phenylpyruvic acid | 156-06-9 | SIGMA | 286958-5G |  | Phenylpyruvic acid derivatives |
| 2-Aminobenzoic acid | 118-92-3 | SIGMA | A89855-25G | Benzene and substituted derivatives | Aminobenzoic acids |
| Urocanic acid | 104-98-3 | SIGMA | 859796-5G | Azoles | Imidazolyl carboxylic acids and derivatives |
| Acetyl-CoA | 102029-73-2 | SIGMA | A2056-10MG | Not classified |  |
| Fumaric acid | 110-17-8 | SIGMA | F8509-100G |  |  |
| L-Malic acid | 97-67-6 | SIGMA | M7397-25G |  |  |
| 3-Hydroxybutyric acid | 306-31-0 | Wako | 085-03571 |  |  |
| Histamine | 56-92-8 | Wako | 081-03551 |  |  |
| Serotonin | 153-98-0 | SIGMA | H9523-25MG |  |  |
| 3-Dehydroshikimic acid | 2922-42-1 | SIGMA | 05616-10MG |  |  |
| Shikimic acid | 138-59-0 | SIGMA | S5375-10MG |  |  |
| trans-Cinnamic acid | 140-10-3 | SIGMA | C80857-5G |  |  |
| Coumarate | 500-05-0 | SIGMA | C85409-25G |  |  |
| Allantoin | 97-59-6 | Wako | 010-01302 |  |  |
| Guanosine monophosphate | 5550-12-9 | TCI Chemicals | G0172 |  |  |
| Picolinic acid | 98-98-6 | TCI Chemicals | P0421 |  |  |

**Supplementary Table 2 Primer list**

**For ChIP-qPCR**

| Hypothalamic neural peptide-coding genes | | | |
| --- | --- | --- | --- |
| Name | Forward | Reverse | Probe ID |
| *CRH* | GAGCAGCCGTCTAAGTTTGC | GTGTTCGCGGACTATTCCTT | #33 |
| *PNOC* | GGTGAAGCAGGCTTGAGAAT | TGCCATCAGACCTGTGACAT | #10 |
| *SST* | GGCTCAGTCCTTCTACTGTCCA | AGCAACAGGGCATGTGTATG | #39 |
| *NPY* | CTCTTGGCGGCTAAATTCC | AGAAGGGCCGTGGACAAT | #6 |
| *GAST* | ACCCAGGGAGCTTGGTTC | GGGAGTTGACCTTCATGGTG | #78 |
| *AVP* | TGGGGCTGTATCTGTTAGGG | AGTGCACCCACCATTAGGG | #30 |
| *OXT* | AGAGCCTCCTCCCACCTG | ATCCAGCCTCCCAGTACCTC | #23 |
| *POMC* | GGGGACTCAGACAATTTTCAA | GGAACTAAAGCCAAGCCAGA | #81 |
| *CARTPT* | GAGGTTCTGGGGATGGTTAAA | TCCCTGTCCCTCCTTTTTG | #44 |
| *CORT* | AGTGGGTCTGGCATGCTG | CAAGGAGGCCCCAAGAAT | #34 |
| *GALP* | AGAGGGGACAGGGCTACG | CCTGTGTCTTCCTTGGTTGTC | #59 |
| *HCRT* | ACGCGAGACCCCACATTAT | GAGCAGCGACAAGAAGCAG | #55 |
| *KISS1* | GCTCTTCGGAGAGGAAACAA | ACGTGGAAATGGGCTATCTG | #7 |
| *AGRP* | GCATAGACAGTCCTGACGACAC | CGCCTGCCTCATTAGCAC | #32 |
| *PENK* | TGGACTGGCTTAGACTTCTGG | GGAAAGGGGCAGGTTCAC | #67 |
| *CCK* | CATGTTGCCCATAGGTTGC | TGCTGCTCCACTTTCTAATCC | #34 |
| *NPVF* | GACCCATAGAGCAAATACAGAGG | TTGACAAACTGGCGTCTCAG | #16 |
| *PDYN* | GCACCAGCCAACACAGAGT | CGGAAGAGAGTCGGAAGATG | #40 |
| *GNRH2* | TACAGTGAGCAAACGGAAGC | CAGGCTGTTCAGAGGTACAGG | #14 |
| *NPW* | CCCTTCTTCTGGGTCATGC | CTGGACAACAAAGAGAGACTGC | #39 |
| *GHRH* | ACGTCTGGTCCCCCTTCT | CGGCTTAGGAAATTCCACTTC | #34 |
| *PRLH* | CCTGATGCCTGTGTGACG | ATCCAAATGAGGAGGACACG | #7 |
| *TRH* | CCCACTCCTGGCAGGTAAC | GGCTCCCTTTCCAGTGCT | #12 |
| Hypothalamic neuron differentiation-related genes | | | |
| *HMX2* | GGAAGGGGAATGTCTGCTC | GGGTGAGGTGGGTTAGGAG | #67 |
| *POU3F2* | GCCAGCATCTCCTTACAGGT | GACTGCGGGGTGTCCTAAG | #58 |
| *RAX* | TTGGAAAGATGATTGGATAGGG | ACAGCGCCATTGTCCATC | #67 |
| *SHH* | CAGCAATCAAAAGACAAAAAGAGT | GCTAAAGGCAGCCTCTCTCA | #80 |
| *OTP* | CAGAGGCACAGAGCTACGC | CGGCTCCCTCTAGTTCTTCC | #80 |
| *BMP7* | ACCTCTCCCCACTCACCAC | TGGCAGGTCTTGGAGGTCT | #52 |
| *ASCL1* | AGGAGCGGGAGAAAGGAAC | CCACCCACTCCCTAAACTCC | #55 |
| *ARNT2* | CATGCTAAGTGGGCAGCA | CCATTTAGATTCAGAGGGAAGC | #14 |
| *NKX2-1* | TCCCTTGGCAGCTTTTCA | GAGAACTGCAGGCCTGGTT | #40 |
| *NR5A1* | GGAGGTAGCCATTCACAAGG | GGAGTGGGGCTTGATTTATG | #32 |
| *SIM1* | TGTTGTTTGGGCAACTTCC | ATGCTGGCATTCCCTCAC | #28 |
| *LHX1* | TCTTACACCCCTAAATGGATGG | GGCCTGTCACCTCCCTCT | #51 |
| *SIX3* | ATGCTGCACTCACTGTGGAT | AGTCAAGATTCAGCGATTCCA | #90 |
| *VAX1* | ATTAACTTTCCTTTTGCCTTTGAC | TATCTGGCGAGTGTGGAGAA | #15 |
| Pan-neural marker genes | | | |
| *TUBB3* | GAAGTATCAGAAGGCCCTTGTTC | CTCCTCCTGGGCCAGATATT | #77 |
| *MAP2* | GTGTGTGTGTCTGGCATTAACA | TCCCCAGGAGTCGAGTCTATC | #54 |

**For RT-qPCR**

| Neural peptide-coding genes | | | |
| --- | --- | --- | --- |
| Name | Forward | Reverse | Probe ID |
| *AGRP* | GCCCCACTGAAGAAGACAACT | CTGAGCCTCCTGCAACAGA | #29 |
| *CARTPT* | CAGCAACGACGAGTTTCAGA | CAGAGGTAGCATCAGCAGCA | #74 |
| *CORT* | TGTTAAATCACTTGTTTTTGTTCTCA | GGAGAAGTTCTGCTTCCTCGT | #4 |
| *CRH* | CAGGGCCCTATGATTTATGC | CGCTCTCTTGACAGCTCGAT | #34 |
| *GALP* | GGCAGAGACTCCAGCATCC | CCTCTTTCCGTCTTGGTCAC | #39 |
| *GAST* | CCAGCCTCTCATCATCGAA | CTTCTTGGACGGGTCTGC | #33 |
| *GHRH* | CATCTTCACCAACAGCTACCG | CTCGCTCTTGGTTGCTCTCT | #1 |
| *GNRH2* | TCCACAGCTCTTCCTTGAGC | CAGTCAGCAGCAGCAGGA | #1 |
| *KISS1* | GGTGGTCTCGTCACCTCAG | CTAGAAGTGCCTTGAGGCTTG | #51 |
| *NPW* | TCCCTGGCACTTCCACTC | CTCCTCTGAGGGGACACG | #13 |
| *NPY* | CGCTGCGACACTACATCAAC | CTCTGGGCTGGATCGTTTT | #9 |
| *OXT* | AGCCTCGCTTGCTGTCTG | GGGGGCAGTTCTGGATGTA | #79 |
| *PDYN* | TGATGCATAAGCACCTCTTTTC | TGAATGCACTCCAACCTGAA | #2 |
| *PENK* | ACATCAACTTCCTGGCTTGC | GCAGGTTTCCCAAATTTTCA | #8 |
| *POMC* | GGGTCGTGGCAGATAATCA | CAGTTTACATTCAAAGTCAGAGGTG | #27 |
| *PRLH* | GCTCCTGTGCCTGCTGAT | GTGCCGATGGGTACGACT | #24 |
| *TRH* | TGAACCTGACCGGTGTCC | CTGGCGCAGGAAGTCATC | #3 |
| Pan-neural marker genes | | | |
| *TUBB3* | ACGTGTGAGCTGCTCCTGT | AAAAACAAAACCGTAAAACGTCA | #1 |
| *MAP2* | CGAACTTTATATTTTACCACTTCCTTG | CCGTTCATCTGCCATTCTTC | #2 |
| Epigenetic factor genes | | | |
| *SETD1A* | CATCGAATACGTGGGTCAGA | AATGCCCTCCTGCACGTA | #7 |
| *SETD1B* | TCAGGTGCTGTCCTCTACCC | ACGACAGAGAAGCCCTTTCC | #1 |
| *ASH1L* | TCTGCACCAAACCCTATGCT | GTTGTCTGGGACGTAATGAGG | #60 |
| *MLL1* | GACAGTGTGCGTTATGTTTGACT | TGGCCAATATATAGTAAACGACCA | #9 |
| *MLL2* | GGAGAACCAGACCATTGTGC | TTCTGAATGGGCGAGTGG | #1 |
| *MLL4* | ACAGGCGGAGGTGTTGAG | GGCAGGTCAGCAGGTATCAC | #44 |
| *SETD7* | GCCCAAAGGGAGTGACTGT | CAACCCACTTGATCGAGGTT | #2 |
| *SMYD3* | CCTGCCTTTGACCTTTTTGA | AGATACTGGGATATAGGCCAACAC | #4 |
| *PRDM9* | TCCTCCTCACTCACCAGAGG | CCACACTCCCTGCAGACATA | #7 |
| *EZH1* | TCAAGATTGAACCAGAACCATGT | GGTTGTGGAGCATGGCATA | #38 |
| *EZH2* | CGCTTTTCTGTAGGCGATGT | TGGGTGTTGCATGAAAAGAA | #38 |
| *SUZ12* | ACATGGGAGACTATTCTTGATGG | GCAACGTAGGTCCCTGAGAA | #16 |
| *KDM1A* | CCATGGTGGTAACAGGTCTTG | TGATCTTGGCCAGTTCCATA | #76 |
| *KDM1B* | CCCGGGTACTCGGTGATAAT | CATCTCCAGAATAATCAATACACTGC | #29 |
| *KDM2B* | TCCACCCTGGATGCCTTA | CCGGCGTGGTTACACTTC | #3 |
| *KDM5A* | GGAGTGGCAAAAATAGGAGGA | TTCTTTGAAAACTGCCACCA | #1 |
| *KDM5B* | CCTCCTAGATTCCAGCAATTCTT | CGTTGTCTCCTCGGGTTCTA | #75 |
| *KDM5C* | ACACCTGGGCCAACAAAGT | GTGCCCTCAGTTCTTCAAGG | #10 |
| *KDM5D* | GAGAACATGGCTCCAGGAAA | CAACACAGGGCCAGTCAAC | #1 |
| *NO66* | TGTGGTCTGGCCATGGAT | TCGTATCTTCCTGGACCTCAA | #1 |
| *KDM6A* | GGGACCAATATTGACCTATCTGA | GCTGATACGACACGCACAA | #4 |
| *KDM6B* | CTGCTGACCTGGCTGGAT | CTACGAGGAGGGGGATGAG | #1 |
| *JHDM1D* | GAATTACGCTCTCGAGTCTTCC | CATGTTTCTCCAGATATCTTTGTGTC | #71 |
| *P300* | TCTTCAGCACCATGGACAGT | GTTGCATACGAGGCCCATAG | #1 |
| *CBP* | CGCAAATGACTGGTCACG | GTTCCCAGTTATTCCCATCTTG | #51 |
| *GCN5* | GGAAAAGTTCCGAGTGGAGA | TCCAGCATGGACAGGAATTT | #35 |
| *TIP60* | CAATGTGGCCTGCATCCTA | CCTTCCACTTTGGAGAGTTCAT | #16 |
| *MOZ* | AAGAAGACATGCCCCTACCC | CTGCCTCTGCTTGCATCTC | #12 |
| *HBO1* | CCTTGCACTGATTTTGTCTGAG | TGGGCTGAGATACGGAAGTC | #1 |
| *HDAC1* | CGGTGCTGGACATATGAGAC | TGGTCCAAAGTATTCAAAGTAGTCA | #81 |
| *HDAC2* | AGGTTCAGGTGATTCTCATGC | TGGTGGTGCACACCTGTACT | #75 |
| *HDAC3* | GACCTATGACAGGACTGATGAGG | GAACTCATTGGGTGCCTCTG | #82 |
| *HDAC8* | CCAACAAATCCTCAACTACATCA | AACACGCTGTCTTCATTATAGGC | #73 |
| *HDAC4* | GTGGTAGAGCTGGTCTTCAAGG | GACCACAGCAAAGCCATTC | #24 |
| *HDAC5* | TTGCCAGCACTGAGGTAAAG | GGCTCCTTTGACTTCGACAA | #1 |
| *HDAC7* | GAACAGTCCATCCCAACAGC | GTGGCTCCTTCCGTCTCC | #1 |
| *HDAC9* | AATGCACAGTATGATCAGCTCAG | GGTCTGTCCTTAGGTCTAAAGGTG | #1 |
| *HDAC6* | CCACACTGGACCAGACTACG | TGCTCGAGGCTAGAGGAGTT | #1 |
| *HDAC10* | ATGACCCCAGCGTCCTTT | CATCTGACTCTCGCAGGAAA | #2 |
| *HDAC11* | ACCATAATGGCGGGGAAG | GATGTCCGCATAGGCACAG | #31 |
| *SIRT1* | TGTACGACGAAGACGACGAC | TTCATCACCGAACAGAAGGTT | #63 |
| *SIRT2* | GCTGCTACACGCAGAACATAGA | CTCCACCAAGTCCTCCTGTT | #1 |
| *OGT* | TGCTTTCCTTCCAAGGGTTA | TGTCTCTCAGCTGCCTCAAA | #1 |
| *MGEA5* | TGGTCTAGCAGGAGAGTTCCA | AAACTTTGGAGGTAGGAGTCAGTG | #83 |
| Internal control gene | | | |
| *ACTB* | AGAGCTACGAGCTGCCTGAC | CGTGGATGCCACAGGACT | #9 |

**Supplementary Table 3 Antibody list**

| Name | Company | Cat. No. | Application  (Final conc.)* |
| --- | --- | --- | --- |
| H3K4me3 | MABI | MABI0304 | ChIP (30 μg/mL) |
| H2AS40Gc (20B2) | Produced in-house^21^ | | |
| GHRH | Novus Biologicals | NBP2-29915 | IF (1 μg/mL) |
| CARTPT | CST | 14437S | IF (1 μg/mL) |
| NPY | Millipore | AB9608 | IF (1 μg/mL) |
| AGRP | Neuromics | MO15105 | IF (1 μg/mL) |
| CRH | abcam | ab8901 | IF (1 μg/mL) |
| TRH | Atlas Antibodies | HPA035595 | IF (1 μg/mL) |
| TUBB3 (TUJ1) | COVANCE | MMS-435P | IF (1 μg/mL) |
| Alexa Fluor 488 goat anti-rabbit IgG (H+L) | Invitrogen | A11034 | IF (1:1000) |
| Alexa Fluor 594 goat anti-mouse IgG (H+L) | Invitrogen | A11032 | IF (1:1000) |

*, IF, immunofluorescence assay; ChIP, chromatin immunoprecipitation assay.
